# Theoretical Analyses of Turgor Pressure during Stress Relaxation and Water Uptake, and after Changes in Expansive Growth Rate When Water Uptake Is Normal and Reduced

**DOI:** 10.3390/plants12091891

**Published:** 2023-05-05

**Authors:** Joseph K. E. Ortega

**Affiliations:** Department of Mechanical Engineering, University of Colorado Denver, Denver, CO 80217-3364, USA; joseph.ortega@ucdenver.edu

**Keywords:** turgor pressure, expansive growth, wall loosening, wall extensibility, hydraulic conductance, biophysical growth equations

## Abstract

Turgor pressure provides the force needed to stress and deform the cell walls of plants, algae, and fungi during expansive growth. However, turgor pressure plays another subtle but equally important role in expansive growth of walled cells: it connects the two biophysical processes of water uptake and wall deformation to ensure that the volumetric rates of water uptake and enlargement of the cell wall chamber are equal. In this study, the role of turgor pressure as a ‘connector’ is investigated analytically by employing validated and established biophysical equations. The objective is to determine the effect of ‘wall loosening’ on the magnitude of turgor pressure. It is known that an increase or decrease in turgor pressure and/or wall loosening rate increases or decreases the expansive growth rate, respectively. Interestingly, it is shown that an increase in the wall loosening rate decreases the turgor pressure slightly, thus reducing the effect of wall loosening on increasing the expansive growth rate. Other analyses reveal that reducing the rate of water uptake results in a larger decrease in turgor pressure with the same increase in wall loosening rate, which further reduces the effect of wall loosening on increasing the expansive growth rate.

## 1. Introduction

Plants, algae, and fungi increase in size predominately by the enlargement of their individual cells, i.e., by expansive growth. Expansive growth is defined as the permanent increase in the volume of the cell, and it is fundamental to the development, morphogenesis, and sensory growth responses of plants, algae, and fungi. The cells of these organisms have walls that provide them with shape, physical and chemical protection, and a surface to interact with other cells and the environment. Research has shown that two biophysical processes are needed for expansive growth: water uptake and wall deformation. During expansive growth, water is absorbed by osmosis. The water uptake and the wall’s resistance to deformation increase the pressure inside the cell. The pressure difference, internal and external to the plasma membrane, is defined as the turgor pressure: *P* = *P*_I_ − *P*_E_. (See Appendix A for definitions and description of individual variables and terms.) A value of *P* greater than zero stresses the wall and deforms it. The wall deformation is both reversible (elastic) and irreversible (plastic). The elastic deformation contributes to the production and maintenance of *P*. The plastic deformation produces a permanent increase in volume of the wall chamber and the shape of the cell. Plastic deformation only occurs when the *P* exceeds a critical value, *P*_C_.

A general model for expansive growth of walled cells is as follows. Water uptake by osmosis increases the volume of the protoplast and produces *P*. The *P* stresses the wall and deforms it. Plastic deformation of the wall enlarges the volume of the wall chamber irreversibly and produces expansive growth. This general model provided the impetus for early experimental studies concerning the measurement of *P* and the mechanical behavior of the wall. In 1965, Lockhart [1] proposed a set of three biophysical equations that describe *steady* expansive growth, i.e., when the expansive growth rate and *P* are constant. These equations provide a quantitative component to the conceptual model and provided the impetus for both experimental, e.g., [2,3], and theoretical research, e.g., [4]. Over time, a shortcoming of both the general conceptual model and the Lockhart equations was revealed; they do not provide an explicit description of how the wall deformation rate and water uptake rate are regulated during expansive growth, especially when the growth rate changes.

In 1985, Cosgrove [5] showed that when water uptake was eliminated from growing plant tissue (and transpiration was suppressed), the *P* decreased exponentially to *P*_C_. The exponential decay in *P* was attributed to the exponential decrease in stress in the wall (stress relaxation) that was produced by “wall loosening”. It was also in 1985 that Ortega [6] augmented the wall deformation equation derived by Lockhart with an elastic term. The elastic term employs the derivative d*P*/d*t* (where *t* is time) and produces a first order differential equation whose solution describes the exponential decay of *P* that was experimentally observed [5]. Subsequently, in 1993, Cosgrove [7,8] postulated a mechanism describing how stress relaxation and water uptake are connected during expansive growth. Essentially, a “stress relaxation–water uptake” (SR-WU) mechanism was postulated to explain how a change in wall stress is coupled to a change in water uptake. *P* was identified and highlighted as a floating biophysical variable that couples changes in wall stress to changes in water uptake. Overall, the SR-WU mechanism provides a conceptual model that connects wall loosening to wall stress relaxation and a decrease in *P*, where the decrease in *P* produces an increase in water uptake. In turn, the water uptake deforms the loosened wall to produce expansive growth. Importantly, a quantitative analysis of the SR-WU mechanism has yet to be conducted. The results of quantitative analyses could provide an additional test of the validity of the SR-WU mechanism and reveal subtle characteristics that may provide additional insight into the *P* behavior during expansive growth.

Here, the SR-WU mechanism is analyzed theoretically and quantitatively. Analyses are conducted with established biophysical equations that have been validated with experimental results (see reviews [9,10]). The analyses provide insight into the SR-WU mechanism and reveal three important biophysical variables that control the magnitude and behavior of *P* during expansive growth: the irreversible wall extensibility (*ϕ*), the volumetric elastic modulus (*ε*), and the hydraulic conductance of the plasma membrane (*L*). The value of *ϕ* is a measure of the rate of ‘wall loosening’ and the irreversible (plastic) wall deformation rate. *ε* is a measure of the reversible (elastic) deformation of the wall, and *L* is a measure of the rate of water uptake into the cell. The analyses show that the time constant, *t*_SR_ = 1/*εϕ*, controls *P* behavior during stress relaxation, and that the time constant, *t*_WU_ = 1/*εL*, controls *P* behavior during water uptake. It is also shown that *ε*, *ϕ*, and *L* control *P* during changes in expansive growth rate and that they contribute to the final magnitude of *P* after changes in expansive growth rate. Other analyses investigate the behavior of *P* during the initiation of expansive growth, and after changes in the magnitude of *ϕ*. The results show that after growth is initiated, a decrease *P* occurs. It is also shown that an increase in the magnitude of *ϕ* during growth produces a decrease in *P*. A decrease in *ϕ* increases *P*. In other analyses, it is shown that the magnitude of the changes in *P* after changes in *ϕ* is dependent on the value of *L*. Smaller rates of water uptake (smaller values of *L*) amplify the decrease in *P* produced by *ϕ*. Small values of *L* can reduce the values of *P* and the expansive growth rate considerably for the same amount of wall loosening, i.e., the same value of *ϕ*. It is suggested that a change in water availability may be simulated analytically by a change in the magnitude of *L*.

## 2. Analyses

### 2.1. Stress Relaxation–Water Uptake (SR-WU) Mechanism 

**Figure 1:**
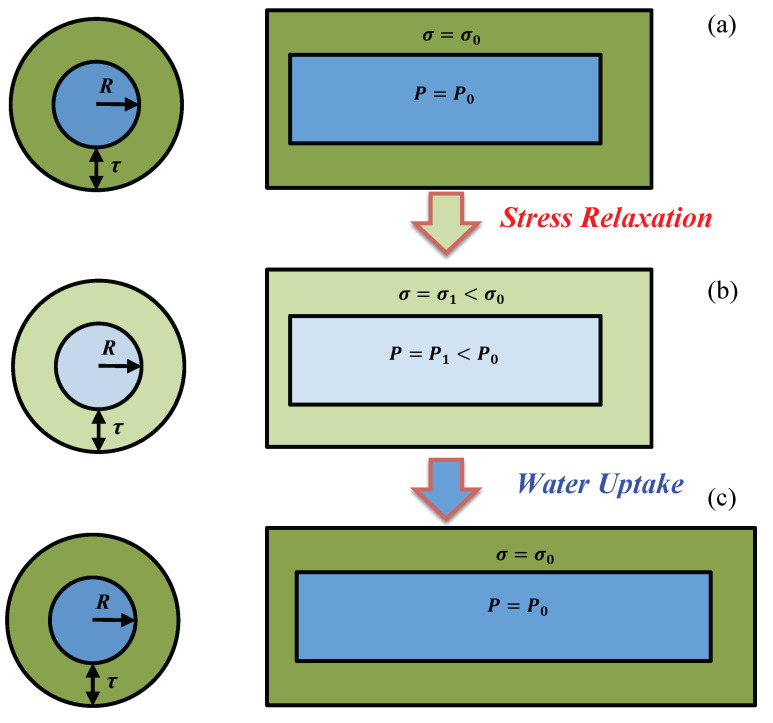
A schematic illustration of the cross-section and side-section of a cylindrical plant cell undergoing the SR-WU mechanism and producing elongation growth. The wall (green) encloses the contents of the cell (blue) and forms a wall chamber. The plasma membrane is considered to be located on the inside surface of the wall chamber (not shown). The SR-WU mechanism is described in three stages. In stage (**a**), the wall is stressed to some initial value, *σ* = *σ*_o_ (dark green), and the initial turgor pressure (dark blue) has a value, *P*_o_ = (2*τ*/*R*) *σ*_o_, where *τ* is the wall thickness and *R* is the radius of the cylinder. The wall is “loosened” and ***stress relaxation*** occurs between stages (**a**,**b**), reducing the magnitudes of the wall stress, *σ*_1_, and turgor pressure, *P*_1_, in stage (**b**). The lighter colors indicate the smaller magnitudes of *σ* and *P*. The smaller *P* in stage (**b**) initiates ***water uptake*** between stages (**b**,**c**). The water uptake increases the length of the loosened wall and restores the wall stress and turgor pressure to their initial values (*σ*_o_ and *P*_o_) in stage (**c**).

Initially, stress relaxation and water uptake that occur during expansive growth were described in a stepwise description for the sake of clarity [7,8], but it was emphasized that they occur concurrently during expansive growth. Here, the SR-WU mechanism is described in a similar stepwise fashion for clarity and because it assists the analyses. The SR-WU mechanism is thought to occur in three stages. Figure 1 shows schematic illustrations of a wall chamber (green) surrounding the protoplast (blue, mostly water) of a plant cell. The wall chamber is considered to be a thin-walled cylinder with a constant radius, *R*, and constant wall thickness, *τ*, that is pressurized by *P* (thick walls are used in the schematic illustration for labeling purposes). The plasma membrane is located on the inner surface of the wall chamber but is not shown. In this description, expansive growth only occurs in the longitudinal direction. In stage (a), the initial longitudinal wall stress, *σ*_o_, produced by the initial turgor pressure, *P*_o_, is *σ*_o_ = (*R*/2*τ*) *P*_o_. It should be noted that the wall stress and turgor pressure are directly related to one another by *R*/2*τ*, so *σ* changes when the *P* changes. Between stage (a) and stage (b), ***stress relaxation*** of the wall occurs. Loosening the wall by breaking load-bearing bonds between polymers within the wall produces stress relaxation that reduces the values of both *σ* and *P*. It is shown that in stage (b), the values *σ*_1_ and *P*_1_ are smaller than their original values; the light green and light blue colors indicate the smaller magnitudes. The smaller *P* initiates ***water uptake*** between stage (b) and stage (c). In stage (c), the water uptake increases the length of the loosened wall and tightens the wall again, restoring *σ* and *P* to their initial magnitudes. The overall result of the stress relaxation and water uptake processes is that the length of the wall chamber has permanently increased, stage (c).

This description of the SR-WU mechanism provides the conceptual framework for quantitative mathematical analyses of the two biophysical processes that culminate in expansive growth, i.e., stress relaxation and water uptake.

### 2.2. Quantitative Mathematical Analyses of the SR-WU Mechanism

Three biophysical equations describing the rate of water uptake, rate of wall deformation, and change in *P* are employed to conduct the analyses (see Appendix B). Governing equations for the isolated behavior of *P* during stress relaxation of the wall chamber and water uptake by osmosis are provided in Appendix C. In Appendix C, the solutions to the respective governing equations are also presented. Equation (1) calculates *P* as a function of time, *t*, for the stress relaxation that occurs between stages (a) and (b) in Figure 1.

(1) $$P\left(t\right)=(P_{o}-P_{C\;})\;\exp\left(-\frac{t}{t_{\mathrm{SR}}}\right)+P_{C\;}$$

Equation (1) describes the exponential decrease in *P* from an initial value, *P*_o_, to the final value, *P*_C_, with a time constant, *t*_SR_ [5,6]. In other words, Equation (1) shows that the time required for the *P* to decrease from *P*_o_ to *P*_C_ during stress relaxation depends on the magnitude of the time constant *t*_SR_. A small value of *t*_SR_ increases the decay rate of *P* and shortens the time required to decrease to *P*_C_. A large value of *t*_SR_ decreases the decay rate of *P* and lengthens the time required to decrease to *P*_C_. It is shown in Appendix C that *t*_SR_ = 1/*εϕ*.

Similarly, the water uptake increases *P* exponentially between stages (b) and (c) in Figure 1; see Appendix C for the governing equation and its solution, *P*(*t*). Equation (2) describes an exponential increase in *P* from *P*_C_ to *P*_o_, with a time constant, *t*_WU_.

(2) $$P\left(t\right)=(P_{C}-P_{o})\;\exp\left(-\frac{t}{t_{\mathrm{WU}}}\right)+P_{o}$$

Equation (2) shows that the time required for *P* to increase from *P*_C_ to *P*_o_ during water uptake depends on the magnitude of the time constant *t*_WU_. A small value of *t*_WU_ results in a faster rise in *P*, and a large value of *t*_WU_ results in a slower rise in *P*. It is shown in Appendix C that *t*_WU_ = 1/*εL*.

It is concluded that the time constants *t*_SR_ and *t*_WU_ control the rate of change of *P* during stress relaxation and water uptake, respectively.

### 2.3. Magnitudes of t_SR_ and t_WU_ for Fungal, Algal, and Plant Cells during Normal Growth

Knowing the values of *t*_SR_ and *t*_WU_ for some representative walled cells can assist the quantitative analysis of the stress relaxation and water uptake processes. Table 1 presents the values of *t*_SR_ and *t*_WU_ for three different species of walled cells growing in normal conditions. It can be seen that for all three species of walled cells, *t*_SR_ is much larger than *t*_WU_, i.e., *t*_SR_ = 1/*εϕ* >> *t*_WU_ = 1/*εL*.

**Figure 2:**
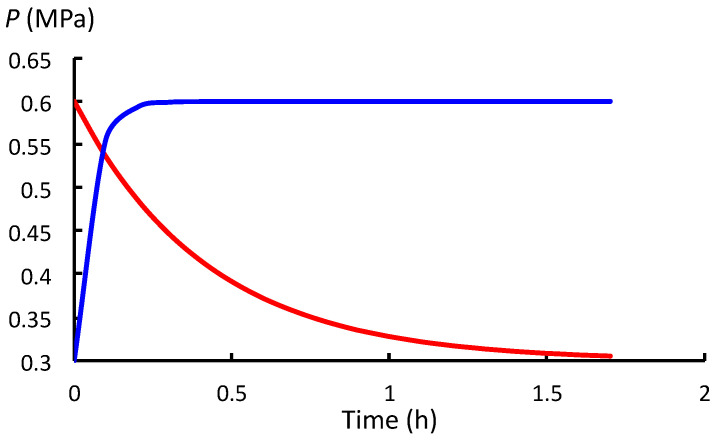
*P*(*t*) between *P*_o_ and *P*_C_ during stress relaxation (red) and subsequently between *P*_C_ and *P*_o_ during water uptake (blue) for cells of growing pea stem tissue (*P. sativum* L.).

**Figure 3:**
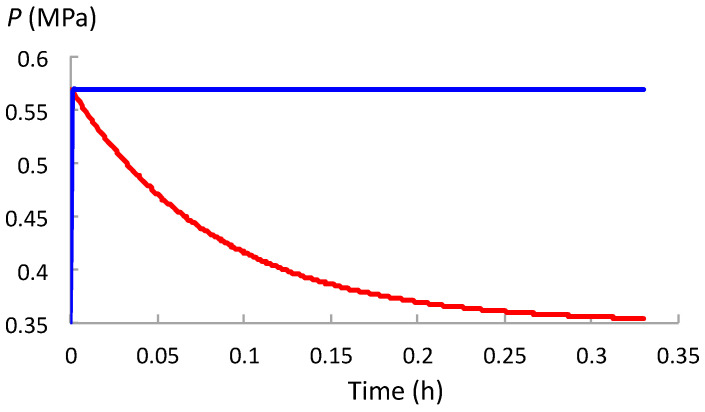
*P*(*t*) between *P*_o_ and *P*_C_ for stress relaxation (red) and subsequently between *P*_C_ and *P*_o_ during water uptake (blue) in internode algal cells of *C. corallina*.

**Figure 4:**
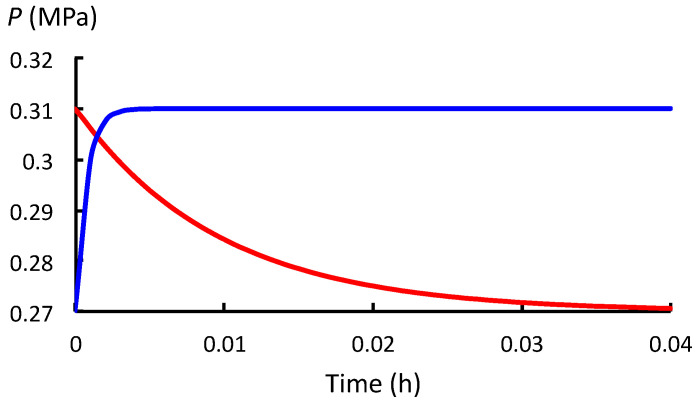
*P*(*t*) between *P*_o_ and *P*_C_ for stress relaxation (red) and subsequently between *P*_C_ and *P*_o_ during water uptake (blue) in stage IV sporangiophores of the fungus *P. blakesleeanus*.

Overall, the values of *t*_SR_ and *t*_WU_ indicate that the *P* decay rate during stress relaxation (between stages (a) and (b) in Figure 1) is smaller (slower) than the rate of increase in *P* during water uptake (between stages (b) and (c) in Figure 1). The difference can be visualized by plotting *P*(*t*) for the respective processes against the same time scale so they can be compared (Figure 2, Figure 3 and Figure 4). Note that in the stepwise SR-WU mechanism, stress relaxation (red curve) occurs first and then water uptake (blue curve) follows to restore *P* to its original value. The respective curves are produced using Equations (1) and (2), and the values for *t*_SR_ and *t*_WU_ presented in Table 1. *P*(*t*) is plotted for cells in growing pea stem tissue of *P. sativum* L. (Figure 2), internode algal cells of *C. corallina* (Figure 3), and stage IV sporangiophores of the fungus *P. blakesleeanus* (Figure 4).

Note the different time scales and different range of *P*_o_ to *P*_C_ for each species of walled cell. It can be seen in Figure 2, Figure 3 and Figure 4 that the time required for *P* to decrease from *P_o_* to *P*_C_ during stress relaxation is considerably longer than the time needed to restore the *P* to its original value during water uptake. Figure 2, Figure 3 and Figure 4 visually demonstrate that the rate of stress relaxation is much slower than the rate of water uptake for all three species of walled cells.

It is concluded that for normal growing walled cells, *t*_SR_ = 1/*εϕ* >> *t*_WU_ = 1/*εL*. This demonstrates that for each biophysical process conducted alone, in isolation, *P* decreases more slowly during stress relaxation compared with its increase during water uptake.

### 2.4. Turgor Pressure during Expansive Growth

During expansive growth, stress relaxation of the wall and water uptake occur concurrently and not in isolation or in a stepwise fashion as was analyzed in the previous sections. However, the values of the biophysical variables (*ε*, *ϕ*, and *L*) for each walled cell species remain constant during steady expansive growth, and the conclusion that stress relaxation and water uptake are controlled by their respective time constants, *t*_SR_ and *t*_WU_, is still correct. Therefore, it would be expected that the time constant *t*_c_ for changes in *P* during expansive growth would include both time constants, *t*_SR_ and *t*_WU_.

During expansive growth, Equation (A3) in Appendix B governs the behavior of *P*(*t*). For walled cells that do not transpire, or where transpiration can be neglected, Equation (3) governs the *P*(*t*) [6]. The osmotic pressure difference across the plasma membrane is Δ*π*.

(3) $$\frac{dP}{dt}=\varepsilon \;\left\{L\;\left(\Delta \pi -P\right)-\varphi \;\left(P-P_{C\;}\right)\right\}$$

Equation (3) is obtained from Equation (A3) in Appendix B by setting the transpiration rate equal to zero, i.e., (d*V*/d*t*)/*V*)_T_ = 0. *P*(*t*) is the solution to Equation (3) [6,16].

(4) $$P\left(t\right)=(P_{o}-P_{\mathrm{eq}\;})\;\exp\left[-\varepsilon \;\left(\varphi +L\right)\;t\right)]+P_{\mathrm{eq}\;}$$

Equation (4) describes the exponential change in *P* from an initial constant value, *P*_o_, to another constant value, *P*_eq_. The time constant for the exponential change is calculated as *t*_c_ = [*ε*(*ϕ* + *L*)]^−1^. It should be noted that 1/*t*_c_ = 1/*t*_RS_ + 1/*t*_WU_ = *εϕ* + *εL* = *ε*(*ϕ* + *L*). *P*_eq_ is obtained by Equation (5).

(5) $$P_{\mathrm{eq}}=\frac{L\;\Delta \pi +\varphi \;P_{C}}{\varphi +L}$$

*P*_eq_ is the new constant equilibrium *P* after the exponential change, and its value depends on the values of *ϕ*, *L*, Δ*π*, and *P*_C_. For any species of walled cell, the steady (constant) relative expansive growth rate, *v*_s_, can be determined using Equation (6) and the value of *P*_eq_.

(6) $$v_{s\;}=\varphi \;(P_{\mathrm{eq}}-P_{C})$$

Equation (6) was first derived by Lockhart [1], and it can be obtained from Equation (A2) in Appendix B for the more limiting case when *P* is constant (then d*P*/d*t* = 0).

### 2.5. Turgor Pressure When ϕ Increases

**Figure 5:**
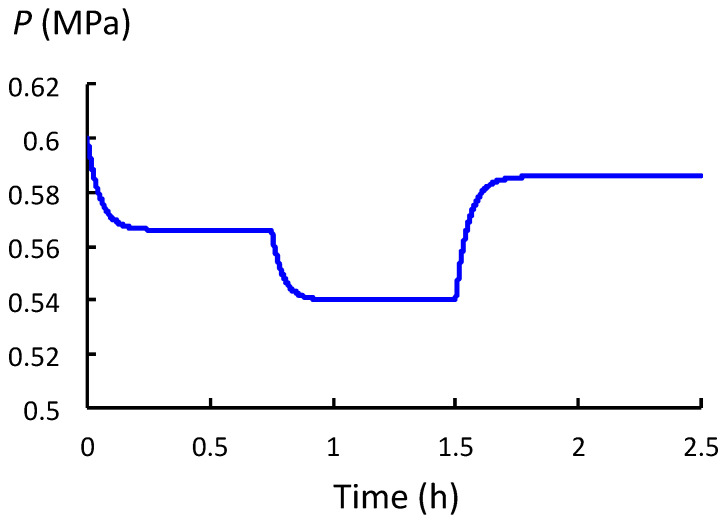
The value of *P* after *ϕ* increases at *t* = 0 h and *t* = 0.75 h, and after *ϕ* decreases at *t* = 1.5 h. See Table 2 for the values of *ϕ*, *P*_eq_, *t*_c_, and *v*_s_ for each time interval.

The relationship between *P* and *ϕ* is investigated during expansive growth by analyzing *P*(*t*) after changes in the value of *ϕ*. *P*(*t*) and *P*_eq_ are determined using Equations (4) and (5), respectively. The steady relative growth rate, *v*_s_, is determined using Equation (6). The analyses use the biophysical properties of a hypothetical but representative plant cell similar to the growing cells in pea stems of *P. sativum* L.; see Table 2 for initial values of the relevant biophysical variables in the column labeled 0.0 h ≤ *t <* 0.75 h. The top row in Table 2 (left to right) shows the time intervals during which different values of *ϕ* are used for the analyses. Initially, the analyses simulate a plant cell that is turgid but not growing (*t* < 0). At *t* ≥ 0, the wall begins to loosen and *ϕ* instantaneously increases from zero to 0.25 h^−1^ MPa^−1^. Immediately, *P* begins to decrease exponentially to a new *P*_eq_ (0.566 MPa), as shown in Figure 5. The new *P*_eq_ is reached in approximately four time constants: 4 *t*_c_ = 4 (0.049 h) = 0.196 h. (See the time interval 0.0 h ≤ *t* < 0.75 h in Table 2 for the calculated values of *P*_eq_, *t*_c_, *t*_RS_, *t*_WU_, and *v*_s_).

Figure 5 is a plot of *P* versus time for all the time intervals analyzed. It can be seen in both Table 2 and Figure 5 that the value of *ϕ* becomes 0.50 h^−1^ MPa^−1^ and 0.10 h^−1^ MPa^−1^ for the two subsequent time intervals 0.75 h ≤ *t <* 1.5 h and 1.5 h ≤ *t <* 2.5 h. The values of *P*_eq_, *t*_c_, *t*_RS_, *t*_WU_, and *v*_s_ for three different values of *ϕ* (0.25 h^−1^ MPa^−1^, 0.50 h^−1^ MPa^−1^, and 0.10 h^−1^ MPa^−1^) are determined and presented in respective columns in Table 2.

In Figure 5, it can be seen that at *t* = 0, when wall loosening starts (*ϕ* = 0.25 h^−1^ MPa^−1^), *P* begins to decrease exponentially from *P* = 6.0 MPa to *P*_eq_ = 0.566 MPa (Table 2). Afterwards, at *t* = 0.75 h, wall loosening increases (*ϕ* = 0.50 h^−1^ MPa^−1^) and *P* again decreases exponentially from *P* = 0.566 MPa to *P*_eq_ = 0.540 MPa. Finally, at *t* = 1.5 h, wall loosening decreases (*ϕ* = 0.10 h^−1^ MPa^−1^), and *P* increases exponentially from *P* = 0.540 MPa to *P*_eq_ = 0.586 MPa.

Inspection of Equation (6) indicates that an increase in *ϕ* increases ***v*_s_**. While *P*_C_ remains constant throughout the analyses, *P*_eq_ decreases after an increase in *ϕ*; see time interval 0.75 h ≤ *t <* 1.5 h in Table 2 and Figure 5. The decrease in *P*_eq_ decreases the driving force for expansive growth and reduces the magnitude of ***v*_s_**; see Equation (6). It is also shown in Table 2 and Figure 5 that a decrease in *ϕ* produces an increase in *P*_eq_; see time interval 1.5 h ≤ *t <* 2.5 h. Finally, it should be noted that the time constant *t*_c_ decreases as *ϕ* increases, and *t*_c_ increases when *ϕ* decreases.

It is concluded that *P* decreases to a new equilibrium value, *P*_eq_, after an increase in the magnitude of *ϕ*. The decrease in *P*_eq_ reduces the magnitude of the increase in growth rate, *v*_s_, produced by the increased value of *ϕ*. For normal growing cells, the decrease in *P*_eq_ and the magnitude of the reduction in *v*_s_ is relatively small.

### 2.6. Turgor Pressure When ϕ Increases and Decreases, and L Is Small

**Figure 6:**
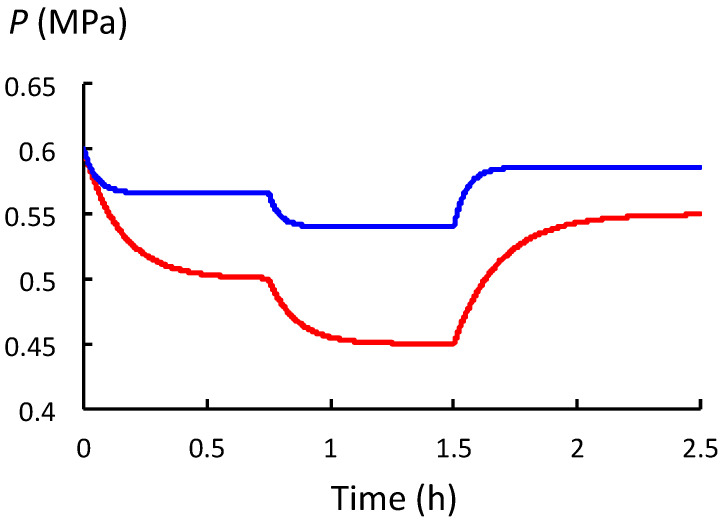
The value of *P* after *ϕ* increases at *t* = 0 h and *t* = 0.75 h, and after *ϕ* decreases at *t* = 1.5 h for two different values of *L*:*L* = 2.0 h^−1^ MPa^−1^ (blue curve), and *L* = 0.5 h^−1^ MPa^−1^ (red curve). For the red curve, see Table 3 for the values of *ϕ*, *P*_eq_, *t*_c_, *t*_RS_, *t*_WU_, and *v*_s_ for each time interval.

The relationship between *P* and *ϕ* is investigated when the value of *L* is decreased. The analysis uses the same biophysical properties of the hypothetical and representative growing plant cell that was used in the previous section (Section 2.5) with one change: the value of *L* is decreased from 2.0 h^−1^ MPa^−1^ to 0.5 h^−1^ MPa^−1^, as shown in Table 3. A decrease in the value of *L* decreases the rate of water uptake. The remainder of the analysis is identical to that conducted in Section 2.5. As before, the values of *P*_eq_, *t*_c_, *t*_SR_, *t*_WU_, and *v*_s_ for different values of *ϕ* are determined using Equations (4)–(6); see Table 3. Figure 6 is a plot of *P* versus time for the time intervals analyzed in Table 3 (red curve). The curve in Figure 5 is also plotted against the same scales in Figure 6 for comparison (blue curve).

A comparison of the two curves in Figure 6 and their respective values (Table 2 and Table 3) reveals several differences. The obvious observation is that the whole curve generated using the small value of *L* (red curve) is shifted to lower values of *P*(*t*). In addition, *P*_eq_ is significantly smaller and *t*_c_ is significantly larger for the same changes in *ϕ* when *L* is small.

It is concluded that *P* decreases to a new equilibrium value, *P*_eq_, after an increase in the value of *ϕ* when *L* is large or small. However, when *L* is small, the value of *P*_eq_ is smaller and *t*_c_ is larger for the same changes in *ϕ*. At smaller values of *L*, larger decreases in *P*_eq_ occur, further reducing the magnitude of the growth rate, *v*_s_, produced by the same value of *ϕ*. For walled cells with a small *L*, the decrease in *P*_eq_ and the magnitude of the reduction in *v*_s_ can be considerable.

## 3. Discussion

### 3.1. Summary

Expansive growth of plant, algal and fungal cells is the culmination of genetic and biochemical processes, and two biophysical processes: deformation of the wall and osmotic water uptake by the cell. During expansive growth, the volumetric rate of water uptake must equal the volumetric rate of enlargement of the wall chamber that encloses the cell. *P* provides the connection between these two biophysical processes. The role of *P* as a ‘connector’ is the topic of this study. The investigation begins with a conceptual model first postulated by Cosgrove [7,8] that describes how stress relaxation of the wall reduces *P* in the cell and, in turn, increases the water uptake. A stepwise description of this model, similar to that described by Cosgrove [7], is presented in a form that is suitable for analysis. The mechanism is termed the stress relaxation–water uptake (SR-WU) mechanism (Section 2.1). The analyses demonstrate that *P* changes exponentially during both stress relaxation and water uptake processes when the biophysical processes are conducted in isolation. It is shown that the exponential change of *P* depends on the value of two time constants: *t*_SR_ = 1/*εϕ* for stress relaxation, and *t*_WU_ = 1/*εL* for water uptake (Section 2.2). In Section 2.3, the values of the relevant biophysical variables (*ε*, *ϕ*, and *L*) for three species of walled cells (plant, algal, and fungal) were used to determine the values of the time constants (Table 1). It is shown that the values of *t*_SR_ are much larger than *t*_WU_ for each species. Figure 2, Figure 3 and Figure 4 show that the relatively large value of *t*_SR_ represents a slow exponential decay of *P* during stress relaxation and the relatively small value of *t*_WU_ represents a fast exponential increase in *P* during water uptake for each species.

Next, the behavior of *P* is investigated during expansive growth, i.e., when both stress relaxation and water uptake occur concurrently. In Section 2.4, the relevant governing equation for *P*, Equation (3), is obtained. Equations (4) and (5) are the solution to Equation (3). It should be noted that the solution describes an exponential change in *P* from an initial constant value, *P*_o_, to another constant value, *P*_eq_; see Equation (4). *P*_eq_ is described by Equation (5). The time constant for the exponential change is defined as: *t*_c_ = [*ε*(*ϕ* + *L*)]^−1^. It is shown that *t*_c_ is related to the time constants previously obtained in Section 2.2 for isolated stress relaxation, *t*_RS_, and isolated water uptake, *t*_WU_, i.e., 1/*t*_c_ = 1/*t*_RS_ + 1/*t*_WU_ = *εϕ* + *εL* = *ε*(*ϕ*+ *L*). In Section 2.5, the behavior of *P* is studied after changes in the value of *ϕ*, i.e., the rate of wall loosening. To conduct this study, a representative hypothetical plant cell similar to the cells in pea stems of *P. sativum* L. is employed; see Table 2. The results of the analysis show that after *ϕ* increases in magnitude, *P* decreases exponentially to a smaller *P*_eq_; see Figure 5 and Table 2. After a decrease in *ϕ*, *P* increases exponentially to a larger *P*_eq_; see Figure 5 and Table 2. It is noted that the value of *t*_c_ decreases when *ϕ* increases and increases when *ϕ* decreases. In the last section, Section 2.6, the value of *L* is decreased and the analytical protocol conducted in Section 2.5 is reproduced; see Table 3. (Note that smaller values of *L* represent smaller rates of water uptake.) It is found that the entire *P*(*t*) curve is lowered to smaller magnitudes when *L* is smaller; see Figure 6 and Table 3. When *L* is smaller, the respective *P*_eq_ values are smaller and the values of *t*_c_ are larger. During normal growth, when *L* is large, it is shown that the expansive growth rates, *v*_s_, produced by an increase in *ϕ* are slightly reduced. The diminished value of *v*_s_ is the result of the decrease in *P*_eq_ produced by *ϕ* (Table 2). When *L* is small, a larger decrease in *P*_eq_ is produced and *v*_s_ is further reduced (Table 3).

### 3.2. Experimental Support

The exponential decay of *P* during isolated stress relaxation, which was analyzed in Section 2.2 and Section 2.3, has been experimentally demonstrated in both plant and fungal cells [5,14]. The exponential rise in *P* during water uptake has been demonstrated in pressure probe methods that are used to determine the hydraulic conductivity of the plasma membrane; see [17] and references within.

The decrease and increase in *P* after respective increases and decreases in the value of *ϕ* have been observed experimentally. A step-up in light intensity elicits a transient increase in the elongation growth rate of the stage IV sporangiophore of *P. blakesleeanus* (light growth response), and the maximum growth rate is nearly twice that of the basal growth rate [18]. An increase in expansive growth rate, *v*, is obtained by increasing the value of *ϕ*; see Equations (6) and (A2). When *P* was continually measured with a pressure probe before and during the light growth response, a small but detectable decrease in *P* was observed during the transient increase in elongation growth rate of the light growth response [19]. The relatively large magnitude of the hydraulic conductance, *L*, of the sporangiophore (Table 1) is probably responsible for the small magnitude of the decrease in *P*.

Other support for the decrease in *P* after an increase in *ϕ* is obtained from studies in the development of the sporangiophores of *P. blakesleeanus*. Stage I sporangiophores exhibit smaller elongation growth rates compared with stage IV sporangiophores [18]. Experimental results demonstrate that *ϕ* is larger and *P*_eq_ is smaller for faster growing stage IV sporangiophores compared with slower growing stage I sporangiophores [20]. In vivo creep experiments determined that the values of *ϕ* for stage IV sporangiophores of “stiff mutants” (C149 and C216) are significantly smaller than those of the wild type. Based on the analyses conducted here (see Table 2 and Figure 5), the *P*_eq_ should be larger for these mutant sporangiophores compared with those of the wild type, and this is confirmed by pressure probe experiments [21].

### 3.3. Wall Loosening (ϕ and P_C_) and Rate of Water Uptake (L)

Wall loosening is defined as the breaking of “load-bearing bonds” between wall polymers in the wall. Continual breaking of load-bearing bonds between wall polymers produces stress relaxation and/or plastic deformation of the wall, or both, depending on how a force is applied to the wall. For example, an increase in force applied to a wall that is growing at a constant rate, increases the plastic deformation rate of the wall and increases the expansive growth rate. This behavior has been observed in growing algal and fungal cells after a step-up in *P* is produced with a pressure probe [11,12,14,20,21]. On the other hand, if an external tensile force, or load, is applied to the growing wall (via a tension and compression machine) and then held constant after a specific load or deformation of the wall is obtained, the applied load decreases exponentially after the initial load is applied, exhibiting stress relaxation. This load behavior has been observed in fungal cells [22,23]. Similarly, if *P* produces the tensile force and the water supply is removed from the growing cell, *P* decreases exponentially, as described in Section 2.2 and Section 2.3. This turgor pressure behavior has been observed in plant and fungal cells [5,14]. The magnitude of the irreversible wall extensibility, *ϕ*, is a measure of the wall loosening in the experiments reviewed above.

However, *ϕ* is not the only biophysical variable that is affected by wall loosening. The value of *P*_C_ is also influenced by wall loosening. Some experimental results, typically from in vivo creep experiments, show that an increase in expansive growth rate is accompanied by an increase in the value of *ϕ* and a decrease in the value of *P*_C_ [20,21]. It was shown that this behavior of *ϕ* and *P*_C_ is a natural result of breaking load-bearing bonds between polymers in the wall [4]. However, in other experiments in which *P* is decreased slowly, as during in vivo stress relaxation experiments [5,14] and when *P* is continually decreased through a series of step-downs [11,12], it is shown that the final value of *P* where growth stops is a constant for each species of cell and independent of the value of *ϕ*. This constant *P* has often been interpreted as *P*_C_. Thus, it appears that the behavior and magnitude of *P*_C_ is related to the method of its determination. Generally, the behavior and determination of *P*_C_ is not resolved, but its variability is relatively small. Therefore, the behavior of *P*_C_ as a function of wall loosening is not addressed in these analyses but will be studied in future research. Here, *P*_C_ is simply considered to be a constant in Equations (5) and (6).

As was previously mentioned, *L* is a measure of the water uptake rate by the cell. The value of *L* reflects the number of open water channels, or aquaporins, and the area of the plasma membrane. In general, larger values of *L* indicate larger water flow rates through the plasma membrane into the cell. Similarly, small values of *L* indicate small water flow rates through the plasma membrane into the cell. Restricted access to water also reduces the water flow rate into the cell. It is apparent that smaller values of *L* simulate restricted access to water, producing water stress or water drought. Therefore, a small value of *L* may be interpreted as a decrease in water availability, and *P* and *v* can be theoretically analyzed during limiting water conditions by varying the magnitude of *L*. Future research will address *P* and *v* in conditions of limited water by varying the magnitude of *L*.

### 3.4. SR-WU Mechanism

The conceptual model described by Cosgrove [7,8] provides a good framework to isolate and evaluate the individual components of stress relaxation and water uptake that occur during expansive growth. The conducted analyses show that in isolation, each component exhibits exponential changes in *P*, and that the rates of change of *P* are controlled by their respective time constants, *t*_SR_ and *t*_WU_. It is shown that *t*_SR_ is much larger than *t*_WU_ for three species of normally growing walled cells (plant, algal, and fungal). This result indicates that the water uptake rate is much faster than the wall stress relaxation rate. This finding is consistent with the results from dimensional analyses, where a dimensionless number, Π_wd_, was derived [13]. This number shows the magnitude of the net water uptake rate was much larger than the wall deformation rate for these same species of walled cells [13]. It is shown that the time constants *t*_RS_ and *t*_WU_, which are identified in the quantitative analyses of the SR-WU mechanism, appear in the time constant *t*_c_ for changes in *P* that occur during expansive growth; see Equation (4) and [16].

### 3.5. P and P_eq_ as a Function of ϕ and L

It is shown here that during expansive growth, the value of *P* exponentially decreases to a smaller equilibrium value, *P*_eq_, after an increase in the value of *ϕ*; see Figure 5. If *ϕ* decreases, *P* exponentially increases to a larger equilibrium value of *P*_eq_ (Figure 5). In both cases, the time constant for the exponential change in *P* is *t*_c_ = [*ε*(*ϕ* + *L*)]^−1^. For normally growing walled cells (when *L* >> *ϕ*), the decrease in *P*_eq_ is small and only reduces the expansive growth rate, *v*_s_, slightly (see Table 2). However, when the values of *L* are smaller and similar to *ϕ*, the values of *P*_eq_ are considerably smaller, and the reduction in expansive growth rate is considerable (Figure 6). In addition, the values of the time constants, *t*_c_, are larger (Table 3) compared with those during normal growth (Table 2). Previously, a similar analysis was conducted [24], and some of the results are comparable with those presented in Figure 5. However, the governing equation, solution, and time constant obtained in [24] are different than those used here.

### 3.6. Molecular Wall Loosening and Irreversible Wall Deformation

The analyses conducted here show that for growing walled cells, an increase or decrease in *ϕ* produces a decrease or increase in *P*, respectively. In Section 3.3, wall loosening was defined as the breaking of load-bearing bonds between wall polymers in the wall. Breaking load-bearing bonds produces irreversible wall deformation. The rate of breaking load-bearing bonds is related to the magnitude of *ϕ*. In general, the molecular biology associated with breaking load-bearing bonds in cell walls is complicated [25,26]. In some plants, enzymes are thought to loosen the wall [27], but in other plants, expansins cause irreversible wall deformation [28]. In some algae, a calcium–pectate cycle is thought to loosen the wall [29]. Additionally, some investigators have found evidence that acidic pH can cause wall loosening in plant walls [30] and fungal walls [31]. A further complication is that dynamic remodeling of the wall many contribute to wall loosening [32]. In the future, one of the significant challenges will be to determine the magnitude of the irreversible wall extensibility, *ϕ*, associated with each of the molecular mechanisms involved in loosening the walls of cells undergoing expansive growth. It is probable that varying combinations of molecular wall-loosening mechanisms are important in cell walls during expansive growth and morphogenesis.

### 3.7. Biological Control of Turgor Pressure?

For a single walled cell undergoing expansive growth, the value of *P* is dependent on the values of Δ*π*, *P*_C_, *L*, *ε*, and *ϕ*. For a walled cell exposed to the atmosphere, the transpiration rate also affects the magnitude of *P*, as shown in Equation (A3). Recent studies suggest that *P* in some plant tissues and specialized cells is under biological control [33]. Specialized cells such as guard cells [34], invasive pollen tubes [35] and invasive fungi [36] appear to exhibit biological control of *P*. Additionally, mutant cells that have abnormal walls seem to biologically regulate *P* in plant cells [37,38] and fungal cells [21,39]. For example, the values of *ϕ* for the walls of stiff mutant stage IV sporangiophores of *P. blakesleeanus* are much smaller than those of the wild type, but the values of *P* for these mutants are larger than those of the wild type [21]. The overall effect is that the elongation growth rates of the stiff mutant and wild type sporangiophores are statistically the same magnitude [21,39].

Biological control of *P* is important for plants and fungi growing in changing environments and climate. The *P* and expansive growth responses of plant and fungal cells to environmental changes can be subtle and complicated. A recent study shows how the biophysical equations, Equations (A1)–(A3), can be used together with relevant experimental methods to determine which biophysical processes are changed, the magnitude of the changes, and their contribution to changes in *P* and expansive growth rate [40]. Water stress is a common condition experienced by many plants and fungi growing in the wild. Future research will focus on how Equations (A1)–(A3), in their dimensional and dimensionless forms, can provide insight into walled cells undergoing water stress.

## Figures and Tables

**Table 1:** Relevant biophysical variables and time constants *t*_SR_ and *t*_WU_ for plant cells in the growth zone of pea stems of *Pisum sativum* L. [5], growing internodes of the algal cells *Chara corallina* [11,12,13], and growing stage IV sporangiophores of the fungus *Phycomyces blakesleeanus* [13,14,15].

Biophysical Variable (Units)	*P. sativum* L. Stem	*C. corallina* Internode	*P. blakesleeanus* Stage IV
*v*_s_ (h^−1^)	0.0751	0.022	0.068
*P* (MPa)	0.60	0.57	0.31
*P*_C_ (MPa)	0.30	0.35	0.27
*L* (h^−1^ MPa^−1^)	2.0	33.0	23.0
*ϕ* (h^−1^ MPa^−1^)	0.25	0.10	1.70
*ε* (MPa)	9.5	120	61
*t*_SR_ = 1/*εϕ* (h)	0.4210	0.0833	0.0082
*t*_WU_ = 1/*εL* (h)	0.0526	0.0003	0.0007

**Table 2:** Values of relevant biophysical variables and calculated values of *P*_eq_, *t*_c_, *t*_RS_, *t*_WU_, and *v*_s_ for three time intervals (top row).

Biophysical Variable (Units)	*t* < 0	0.0 h ≤ *t <* 0.75 h	0.75 h ≤ *t <* 1.5 h	1.5 h ≤ *t <* 2.5 h
Δ*π* (MPa)	0.6	0.6	0.6	0.6
*P*_C_ (MPa)	---	0.3	0.3	0.3
*ε* (MPa)	9.0	9.0	9.0	9.0
*L* (h^−1^ MPa^−1^)	2.0	2.0	2.0	2.0
*ϕ* (h^−1^ MPa^−1^)	0.0	0.25	0.50	0.10
*P*_eq_ (MPa)	0.60	0.566	0.540	0.586
*t*_c_ = 1/(*εϕ* + *εL*) (h)	---	0.049	0.044	0.053
*t*_SR_ = 1/*εϕ* (h)	---	0.444	0.222	1.111
*t*_WU_ = 1/*εL* (h)	---	0.056	0.056	0.056
*v*_s_ (h^−1^)	0.0	0.067	0.120	0.029

**Table 3:** Values of relevant biophysical variables used in Figure 6 (red curve). The values of the respective biophysical variables are the same as in Table 2, except that *L* = 0.5 h^−1^ MPa^−1^.

Biophysical Variable (Units)	*t* < 0	0.0 ≤ *t <* 0.75 h	0.75 h ≤ *t <* 1.5 h	1.5 h ≤ *t <* 2.5 h
Δ*π* (MPa)	0.60	0.6	0.6	0.6
*P*_C_ (MPa)	---	0.3	0.3	0.3
*ε* (MPa)	9.0	9.0	9.0	9.0
*L* (h^−1^ MPa^−1^)	0.5	0.5	0.5	0.5
*ϕ* (h^−1^ MPa^−1^)	0.0	0.25	0.50	0.10
*P*_eq_ (MPa)	0.60	0.500	0.450	0.550
*t*_c_ = 1/(*εϕ* + *εL*) (h)	---	0.148	0.111	0.185
*t*_SR_ = 1/*εϕ* (h)	---	0.444	0.222	1.111
*t*_WU_ = 1/*εL* (h)	---	0.222	0.222	0.222
*v*_s_ (h^−1^)	0.0	0.050	0.075	0.025

## Data Availability

Data sharing not applicable.

## Appendix A. Definitions and Description of Individual Variables and Terms

=	*equal to*
>	*greater than*
>>	*much greater than*
<	*less than*
<<	*much less than*
≥	*greater than or equal to*
≤	*less than or equal to*
A	= *area of the plasma membrane* (m^2^)
*A*_c_	= *cross-section area of a cylindrical cell* (m^2^)
$L_{P}$	=hydraulic conductivity *of the plasma membrane* (m h^−1^ MPa^−1^)
L	$=\left(\frac{L_{p\;}A}{\;V}\right)=relative\;hydraulic\;conductance\;of\;the\;plasma\;membrane$ (h^−1^ MPa^−1^)
P	=turgor pressure (gage pressure relative to the atmosphere) (MPa)
$P_{A}$	=pressure in apoplast, i.e., cell wall (gage pressure) (MPa)
$P_{C}$	=critical turgor pressure (to be exceeded before plastic extension begins) (MPa)
$P_{E}$	=pressure external to the plasma membrane (MPa)
$P_{I}$	=pressure internal to the plasma membrane (MPa)
*R*	= *radius of the cylindrical cell* (m)
t	= *time* (h)
V	= *volume* (m^3^)
$V_{\mathrm{cw}}$	=volume *of the cell wall chamber* (m^3^)
$V_{w}$	=volume *of water in the cell* (m^3^)
$V_{T}$	=volume *of water lost through transpiration* (m^3^)
$v_{\;}$	$=\left(\frac{dV_{\;}}{V_{\;}\;dt}\right)=relative\;rate\;of\;change\;in\;volume\;of\;the\;cell\;$(h^−1^)
$v_{\mathrm{cw}}$	$=\left(\frac{dV_{\mathrm{cw}}}{\;V\;dt{\;}_{\;}}\right)=relative\;rate\;of\;change\;in\;volume\;of\;the\;cell\;wall\;chamber$ (h^−1^)
$v_{s\;}$	$=\left(\frac{dV_{\;}}{\;\;V_{\;}\;dt{\;}_{\;}}\right)=steady\;relative\;rate\;of\;change\;in\;volume\;of\;the\;cell$ (h^−1^)
$v_{T}$	$=\left(\frac{dV_{T}}{\;V\;dt{\;}_{\;}}\right)=relative\;rate\;of\;change\;in\;water\;volume\;lost\;via\;transpiration$ (h^−1^)
$v_{w}$	$=\left(\frac{dV_{w}}{\;V\;dt{\;}_{\;}}\right)=relative\;rate\;of\;change\;in\;water\;volume\;in\;the\;cell$ (h^−1^)
ε	=volumetric elastic modulus of the cell wall (MPa)
ϕ or φ	=irreversible extensibility of the cell wall (h^−1^ MPa^−1^)
π	=osmotic pressure (MPa)
$\pi_{i}$	=internal osmotic pressure or osmotic pressure of the cell sap (MPa)
Δπ	=osmotic pressure difference (MPa)
Π	=dimensionless number or nondimensional number (no units)
L (Δπ−P)	=relative volumetric rate of water uptake (h^−1^)
$\varphi \;\left(P-P_{C}\right)$	=relative volumetric plastic deformation rate of the cell wall (h^−1^)
$\frac{dP_{\;}}{\;\varepsilon {\;}_{\;}dt{\;}_{\;}}$	=relative volumetric elastic deformation rate of the cell wall (h^−1^)

## Appendix B. Biophysical Equations for Expansive Growth

Three biophysical equations were derived, validated with experimental results, and reviewed [9,10]. Equation (A1) describes the relative rate of change in the volume of water in the cell as the difference in osmotic water uptake rate and the transpiration rate [41]. The first term on the right-hand side was first derived by Dainty [42].

(A1) $$v_{w}=L\;\left(\Delta \pi -P\right)-v_{T}$$

*Rate of change in water volume = osmotic water uptake rate—transpiration rate*

Here, *v*_T_ is the rate of relative volume of water lost through transpiration calculated as: *v*_T_ = ((d*V*/d*t*)/*V*)_T_, where *V* is the volume of the cell. Equation (A2), referred to as the “Augmented Growth Equation” when it was first derived [6], describes the relative rate of change in volume of the cell wall chamber as the sum of the plastic and elastic deformation rates of the wall. The first term on the right-hand side was first derived by Lockhart [1].

(A2) $$v_{\mathrm{cw}}=\varphi \;\left(P-P_{C}\right)+\left(\frac{1}{\varepsilon }\right)\;\frac{\;dP}{dt}$$

*Rate of change in cell wall chamber volume = plastic deformation rate + elastic deformation rate*

Recently, an equation was derived and called the “Lockhart Differential Equation” [43], but it is nearly identical to Equation (A2), the “Augmented Growth Equation” that was derived more than three decades ago [6]. Equation (A1) and Equation (A2) both have terms where *P* is explicit. This demonstrates that *P* couples the two equations. Physically, this demonstrates the interdependence of the two processes of wall deformation and water uptake during expansive growth. A third equation describing the rate of change of the turgor pressure, d*P*/d*t*, is obtained by recognizing that the relative rate of change in the volume of the wall chamber equals that of the water uptake [6,9,10,13,16,40] and is shown in Equation (A3).

(A3) $$\frac{dP}{dt}=\varepsilon \;\left\{L\;\left(\Delta \pi -P\right)-{\left(\frac{1}{V}\;\frac{dV}{dt}\right)}_{T}-\varphi \;\left(P-P_{C\;}\right)\right\}$$

*Rate of change in turgor pressure = ε {relative rate of water uptake − relative rate of transpiration—relative rate of plastic deformation of the wall}*

The turgor pressure, *P*, is the pressure difference across the plasma membrane, where *t* is time, *ε* is the volumetric elastic modulus, *L* is the relative hydraulic conductance, Δ*π* is the osmotic pressure difference across the plasma membrane, *V* is the cell volume, ((d*V*/d*t*)/*V*)_T_ is the rate of relative volume of water lost through transpiration, *ϕ* is the irreversible wall extensibility, and *P*_C_ is the critical turgor pressure. The general solution, *P*(*t*), is obtained and presented in [16].

## Appendix C. Governing Equations for Isolated Processes of Stress Relaxation and Water Uptake, and Their Solutions, *P*(*t*)

### Appendix C.1. Governing Equation for Stress Relaxation and Its Solution

Equation (A3) can be used to obtain the governing equation for stress relaxation. The governing equation for stress relaxation is obtained by eliminating the terms for water uptake, *L* = 0, and transpiration, ((d*V*/d*t*)/*V*)_T_ = 0. Then Equation (A4) is obtained [5,6].

(A4) $$\frac{dP}{dt}=-\varepsilon \;\varphi \;\left(P-P_{C\;}\right)\;$$

The solution to Equation (A4) is Equation (A5), where the initial condition, *P*(0) = *P*_o_, is employed.

(A5) $$P\left(t\right)=(P_{o}-P_{C\;})\;\exp\left(-\varepsilon \;\varphi \;t\right)+P_{C\;}=(P_{o}-P_{C\;})\;\exp\left(-\frac{t}{t_{SR}}\right)+P_{C\;}$$

Equation (A5) describes an exponential decrease in *P* from *P*_o_ to *P*_C_ with a time constant, *t*_SR_ = 1/*εϕ*. It is apparent that the time required for *P* to decrease to *P*_C_ depends on the value of the time constant *t*_SR_. A small value of *t*_SR_ increases the decay rate of *P*, and a large value of *t*_SR_ decreases its decay rate.

### Appendix C.2. Governing Equation for Water Uptake and its Solution

The governing equation for water uptake is obtained from Equation (A3) by eliminating the terms for transpiration, ((d*V*/d*t*)/*V*)_T_ = 0, and plastic wall deformation rate (*ϕ* = 0). Then Equation (A6) is obtained, where Δ*π* is replaced by *P*_o_, and *P*_o_ ≤ Δ*π*.

(A6) $$\frac{dP}{dt}=\varepsilon \;L\;\left(P_{o}-P\right)\;$$

The solution to Equation (A6) is Equation (A7), where the initial condition *P*(0) = *P*_C_ is employed.

(A7) $$P\left(t\right)=(P_{C}-P_{0\;})\;\exp\left(-\varepsilon \;L\;t\right)+P_{o\;}=(P_{C}-P_{o\;})\;\exp\left(-\frac{t}{t_{\mathrm{WU}}}\right)+P_{o\;}$$

Equation (A7) describes an exponential increase in *P* from *P*_C_ to *P*_o_ with a time constant *t*_WU_ = 1/*εL*. It is shown that the time required for *P* to increase from *P*_C_ to *P*_o_ depends on the value of the time constant *t*_WU_. A small value of *t*_WU_ results in a faster rise in *P*(*t*), and a large value of *t*_WU_ results in a slower rise.
